# Design of a Wideband Tonpilz Transducer Comprising Non-Uniform Piezoceramic Stacks with Equivalent Circuits

**DOI:** 10.3390/s21082680

**Published:** 2021-04-10

**Authors:** Seonghun Pyo, Muhammad Shakeel Afzal, Youngsub Lim, Seungjin Lee, Yongrae Roh

**Affiliations:** 1 School of Mechanical Engineering, Kyungpook National University, Daegu 41556, Korea; nasgool@naver.com (S.P.); shakeel@gmail.com (M.S.A.); 2 Maritime Integrated Security Systems, LIG Nex1 Co. Ltd., Seongnam 13488, Korea; youngsub.lim@lignex1.com (Y.L.); seungjin.lee@lignex1.com (S.L.)

**Keywords:** multimode Tonpilz transducer, non-uniform drive section, equivalent circuit method, flexural vibration mode

## Abstract

Tonpilz transducers are desirable for their superior performance in underwater target detection and communication applications. Several design schemes to widen their bandwidth have been reported, but these schemes often involve a complex structure or arrangement of additional components. In this study, a simple design is proposed to improve the bandwidth of a multimode Tonpilz transducer by using a non-uniform drive section that consists of piezoelectric stacks of various thicknesses. The efficacy of the design is illustrated with a multimode Tonpilz transducer having three lead zirconate titanate (PZT) stacks of different thicknesses. A new equivalent circuit was developed to analyze the frequency response of the transducer incorporating the non-uniform drive section and was used for rigorous analysis of the effects of varying the position and thickness of the non-uniform stacks on the transmitting characteristics of the transducer. The validity of the design was verified through the fabrication and characterization of a prototype multimode Tonpilz transducer. The developed structure can be readily extended to an arbitrary number of stacks in the Tonpilz transducer with any number of PZT disks in each stack.

## 1. Introduction

Underwater acoustic transducers are used for detecting and tracking submerged objects [1]. In almost all applications, broadband operation of the transducers is desirable for high-speed data transmission and reception [2,3]. Among these underwater transducers, the multimode Tonpilz transducer is the most popular type because of its wide bandwidth characteristics and compact structure [4]. In a typical multimode Tonpilz transducer, the fundamental longitudinal and flexural modes of vibration of its radiating head mass are coupled; in contrast, a single-mode transducer makes use of the longitudinal mode only [5,6,7]. In addition to the typical multimode structure, several other designs have been proposed in the past to widen the bandwidth of the Tonpilz transducer. Rodrigo proposed a wideband transducer design that installs three compliant rods and an additional head mass to combine two resonant modes [8]. Butler [9] and Zhang et al. [10] both proposed multi-resonance structures with additional head masses and compliant layers. Saijyou and Okuyama proposed installing a bending piezoelectric disk in the head mass to widen the bandwidth [11]. Roh et al. incorporated a cavity inside a head mass to reduce the mechanical quality factor of the transducer [12,13]. Butler et al. designed a hybrid magnetostrictive/piezoelectric Tonpilz transducer, which improve coupling coefficient and a wideband doubly resonant response [14]. He et al. designed a new structure of the head mass of a Tonpilz transducer with a single hole to obtain a lower mechanical quality factor [15]. The effects of drive section characteristics such as the poling direction, number of disks, and material properties on the frequency response of the Tonpilz transducer have also been considered. Thompson et al. analyzed the performance of segmented piezoelectric stacks by using materials with a high electromechanical coupling coefficient [16]. Other techniques for increasing the bandwidth such as acoustic impedance matching and electrical impedance matching have also been studied [17,18].

The above techniques for performance improvement of a Tonpilz transducer involve either a significantly complex structure with multiple matching and compliant layers or an additional driving mechanism. In the present study, a simple method is proposed for improving the transmitting characteristics of a multimode Tonpilz transducer by using a non-uniform drive section. A typical Tonpilz transducer has a drive section comprising multiple piezoceramic disks of a uniform thickness. However, the Tonpilz transducer proposed in this work uses piezoceramic disks of different thicknesses. A drive section comprising non-uniform piezoceramic disks is potentially favorable to improve the bandwidth of the Tonpilz transducer as previously reported in a preliminary study of the authors, which checked the feasibility of using the non-uniform drive section in a Tonpilz transducer [19]. Aneela et al. reported the effect of nonuniform layer thickness on the frequency response of a high-frequency ultrasonic transducer as that the nonuniform structure generates even and odd harmonics, which could be utilized to widen the bandwidth [20,21]. However, their research was limited to the vibration of a piezoelectric stack operating in a pure thickness mode, which is different from the multimode Tonpilz transducer in this work operating in both longitudinal and flexural modes [4]. The technique to use a composite electrode configuration to generate even harmonics was introduced by Butler’s, but it was also good only for a transducer operating in a pure longitudinal mode [22].

The acoustic performance of Tonpilz transducers can be analyzed by various methods, such as the theoretical method, equivalent circuit method (ECM), and finite element method (FEM) [23]. The ECM is a simple and efficient technique for analyzing the acoustic characteristics of a Tonpilz transducer [2,23]. Crombrugge and Thompson proposed a systematic approach for using the ECM to optimize a wideband Tonpilz transducer [24]. Various other studies have used the ECM to accurately estimate the performance of a multimode Tonpilz transducer for broadband applications [25,26]. However, the previously reported equivalent circuits cannot be used to analyze Tonpilz transducers comprising non-uniform piezoceramic stacks. Therefore, this study also proposes a new equivalent circuit approach to accommodate the variable thickness of the constituent disks in a piezoceramic stack.

This study presents a new simple structural design method to improve the band-width of a multimode Tonpilz transducer by incorporating a drive section with non-uniform piezoceramic stacks. The efficacy of the design is illustrated with a multimode Tonpilz transducer having three lead zirconate titanate (PZT) stacks of different thicknesses. A new equivalent circuit is developed to represent the non-uniform structure of the drive section and is used to thoroughly investigate the structural effect of the non-uniform piezoceramic stacks on the frequency response of the transducer. Finally, a prototype multimode Tonpilz transducer of practical interest is fabricated to incorporate the non-uniform drive section and is characterized experimentally to validate the design of the non-uniform structure.

## 2. Equivalent Circuit of the Multimode Transducer

**Figure 1:**
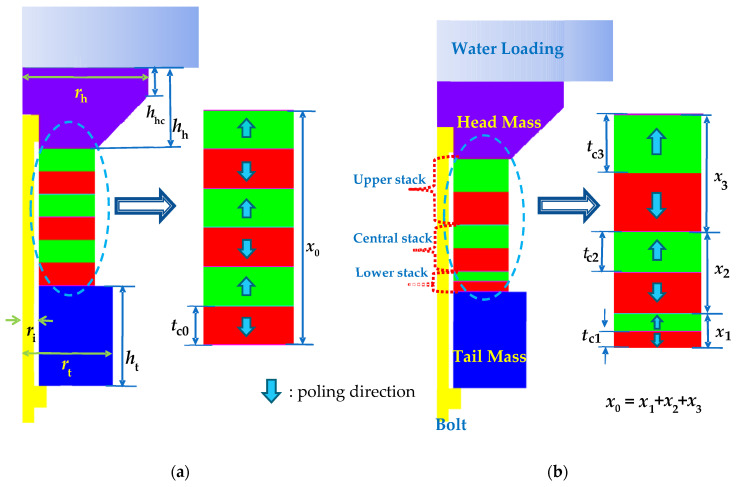
Simplified 2D model of the multimode Tonpilz transducer: (**a**) uniform drive section; (**b**) non-uniform drive section.

Typical multimode Tonpilz transducer structures with uniform and non-uniform drive sections are illustrated in Figure 1 [4,6]. To analyze the structural effect of the drive section more conveniently, a simplified Tonpilz model with six disks was considered initially, as shown in Figure 1. The main components of a transducer are the head mass, drive section, and tail mass, as well as a metallic bolt to fasten all of the components. The material properties of all the non-piezoceramic components are given in Table 1. The drive section comprised the piezoceramic PZT-5H, and its material constants were taken from Butler and Sherman [23]. In Figure 1a, the transducer with the uniform drive section had a stack of six PZT disks, where each disk had a thickness of *t*_c__0_ = 3.33 mm. In Figure 1b, the transducer with the non-uniform drive section had a stack of disks with three different thicknesses of *t*_c1_ = 2 mm, *t*_c2_ = 3 mm, and *t*_c3_ = 5 mm. The non-uniform drive section in this simplified model was divided into three parts. The non-uniform drive section could be divided into two parts, three parts or more. The division into three parts was just a representative case. The front radiating surfaces of both transducers were loaded with water. The total thickness of the PZT stack was kept constant at *x*_0_ = 20 mm for both transducers. The drive sections were arranged so that the poling direction of each disk was opposite to that of its neighboring disks, and all of the disks were connected electrically in parallel and mechanically in series. The non-uniform drive section was divided into three stacks: lower, central, and upper. The initial thicknesses of the lower, central, and upper PZT stacks were *x*_1_ = 4 mm, *x*_2_ = 6 mm, and *x*_3_ = 10 mm, respectively. The outer radius of the PZT stacks was set to *r*_c_ = 12 mm. The thicknesses of the head and tail masses were *h*_h_ = 11 mm and *h*_t_ = 14 mm, respectively, while the outer radii were *r*_h_ = 21 mm and *r*_t_ = 15 mm, respectively. All components had an inner radius of *r*_i_ = 2.75 mm, and the tapped edge of the head mass was *h*_hc_ = 4.0 mm thick. These dimensions were selected by the preliminary analysis to show the clear longitudinal and flexural modes in the transmitting voltage response (TVR) spectrum of the multimode Tonpilz transducer.

Equivalent circuits have been used in many previous works to model typical single-mode Tonpilz transducers. The equivalent circuit can be extended to a multimode Tonpilz transducer by adding suitable circuit elements [26,27]. The multimode Tonpilz transducer in Figure 1 can be represented by the T-network circuit, where each T-branch corresponds to a component of the transducer.

**Figure 2:**
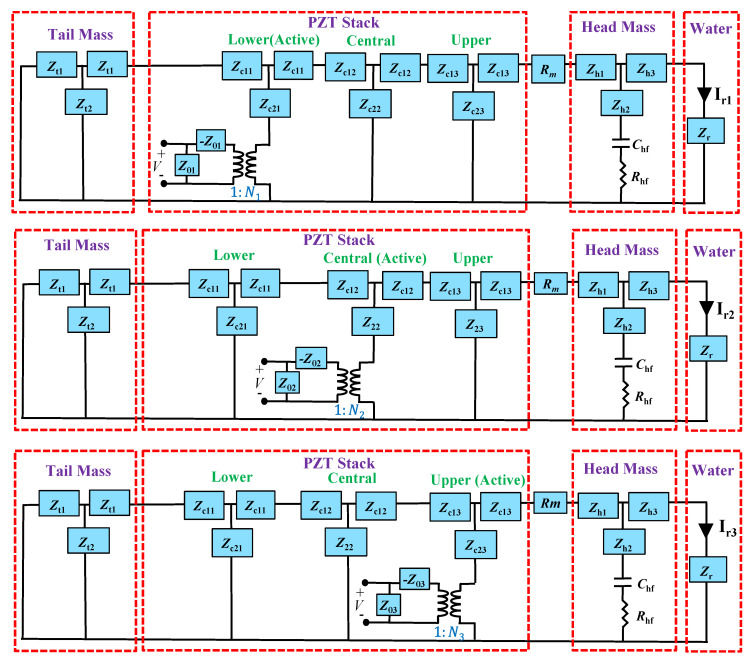
Equivalent circuits for the multimode Tonpilz transducer.

In the case of a uniform drive section, the whole drive section can be described with a general single T network because the static capacitances and electric fields (*E*) of all PZT disks are identical [2]. For a non-uniform drive section, however, the electrostatic capacitance and electromechanical impedance terms differ for each stack. For a given excitation signal, the response of each stack should differ from that of another stack. To incorporate the effect of the non-uniformity, three separate equivalent circuits were constructed as shown in Figure 2, each corresponding to the excitation of a stack in the non-uniform drive section. The first, second, and third circuits in Figure 2 correspond to the Tonpilz transducer in Figure 1 when only the lower, central, or upper stack, respectively, is activated while the other two stacks remain inactive. The inactive two stacks do not operate as piezoelectric stacks but as simple dielectric ceramics. Thus, the property of the activated stack in each circuit is described by stiffened material constants while that of the other two stacks in that circuit is described by unstiffened material constants [28]. The total response to the simultaneous excitation of the three stacks was obtained by summing the responses from the three circuits by the superposition rule. The three circuits in Figure 2 can also be used to estimate the response of the uniform drive section model by setting *x*_1_ = *x*_2_ = *x*_3_.

For the circuits in Figure 2, the terms *Z*_0m_ and *N*_m_ represent the electrical impedance and turning ratio, respectively, of a PZT stack. The subscript *m* is 1, 2, and 3 for the lower, central, and upper stack, respectively, in the non-uniform drive section. In case of the uniform drive section, the electrical impedances and turning ratios are same as *Z*_01_ = *Z*_02_ = *Z*_03_ and *N*_1_ = *N*_2_ = *N*_3_. The circuit parameters *C*_0m_, *Z*_0m_, and *N*_m_ can be calculated as follows:

(1) $$C_{0m}=\left(\frac{\varepsilon_{33}^{S}A_{c}}{t_{cm}}\right)$$

(2) $$Z_{0m}=\frac{1}{j\omega nC_{0m}}$$

(3) $$N_{m}=d_{33}A_{c}/t_{cm}s_{33}^{E},$$

where *t*_cm_ is the thickness of a single disk in the *m*^th^ stack, *ω* is the angular frequency, *A_c_* is the area of the PZT disk, and *n* is the number of PZT disks in the active stack. For the material constants of the piezoceramic, $\varepsilon_{33}^{S}$ is the dielectric permittivity at a constant strain, *d*_33_ is the piezoelectric constant, and $s_{33}^{E}\;$is the compliance at a constant electric field. *Z*_c1m_ and *Z*_c2m_ are mechanical impedances of the PZT disks and are calculated with Equations (4) and (5), respectively. The mechanical impedances of the tail mass *Z*_t1_ and *Z*_t2_ are given by Equations (6) and (7), respectively. *R*_m_ represents the mechanical loss in the longitudinal vibration of the transducer. The radiation impedance (*Z*_r_) imposed on the head mass is given by Equation (8) [29]. The radiation impedance is assumed to be that of a pure piston source for simplification considering that the longitudinal mode is the dominant vibration mode of the head mass over most of the frequency range of interest [7].

(4) $$Z_{c1m}=j\rho_{c}v_{c}A_{c}\tan\left(\frac{k_{c}x_{m}}{2}\right)$$

(5) $$Z_{c2m}=-j\rho_{c}v_{c}A_{c}/\sin\left(k_{c}x_{m}\right)$$

(6) $$Z_{t1}=j\rho_{t}v_{t}A_{t}\tan\left(\frac{k_{t}t_{t}}{2}\right)$$

(7) $$Z_{t2}=-j\rho_{t}v_{t}A_{t}/\sin\left(k_{t}t_{t}\right)$$

(8) $$Z_{r}=R_{r}+jX_{r}=\rho_{w}v_{w}A_{h}[\left(1-2J_{1}\;\left(2k_{w}r_{h}\right)/2k_{w}r_{h}\right)\;+j2H_{1}\;\left(2k_{w}r_{h}\right)/2k_{w}r_{h}].$$

Here, *A*, *ρ*, *v*, *k*, and *r* are the area, density, sound velocity, wave number, and radius, respectively, of a transducer component. The subscripts *c*, *h*, *t*, and *w* correspond to the piezoceramic, head mass, tail mass, and water, respectively. The term *x_m_* is the thickness of stack *m*. *R*_r_ and *X*_r_ are the radiation resistance and reactance, respectively, and *J*_1_ and *H*_1_ are the first-order Bessel and Hankel functions, respectively.

To incorporate the flexural vibration of the head mass, the capacitor *C*_hf_ and resistor *R*_hf_ were added to the head mass branch of the network, as shown in Figure 2 [27]. *C*_hf_ and *R*_hf_ correspond to the mechanical compliance and damping, respectively, for flexure of the head mass of the Tonpilz transducer. The mass term corresponding to the head mass flexure is represented by the inductive term in *Z*_h2_. The head mass has multiple modes of vibration: longitudinal and flexural. The multimode vibration is expressed by the combination of the mechanical impedances *Z*_h1_, *Z*_h2_, and *Z*_h3_ along with *C*_hf_ and *R*_hf_. The three T-branch impedances are expressed as *Z*_h1_ = *jωM*_h1_, *Z*_h2_ = *jωM*_h2_, and *Z*_h3_ = *jωM*_h3_, where the masses *M*_h1_, *M*_h2_, and *M*_h3_ are distributed forms of the total head mass (*M*_h_). *M*_h_ is easily obtained from the density and volume of the head mass. However, the exact values of the three distributed masses are not readily available. Hence, statistical regression analysis of the TVR spectrum of the multimode Tonpilz transducer was used to estimate the values of *M*_h1_, *M*_h2_, and M_h3_ along with the other flexural-mode circuit parameters *C*_hf_ and *R*_hf_. A detailed procedure for estimating these circuit parameters is described in Section 3.

For each circuit in Figure 2, a unit voltage was applied for excitation of respective drive section. The current flowing to the radiation impedance corresponds to the acoustic volume velocity when the PZT stack is excited by the input voltage. The total current *I*_r_ when all PZT stacks are excited simultaneously is obtained by using the superposition rule; the three currents *I*_1_, *I*_2_, and *I*_3_ from each circuit are added to find the total current *I*_r_ to the radiation impedance *Z*_r_. The total current corresponds to the resultant volume velocity *U*_r_ transmitted by the transducer. Subsequently, the radiated acoustic power and sound pressure *P*_r_ can be computed from the volume velocity. The total radiated sound pressure is the sum of all radiated pressures from each circuit, which are represented as *P*_r1_, *P*_r2_, and *P*_r3_. The radiated acoustic power *W* and directivity *D* of the transducer can be calculated with Equations (9) and (10). The directivity is assumed to be that of a pure piston source for simplification as was done with the radiation impedance of the transducer. Then, the TVR level of the Tonpilz transducer can be calculated with Equation (11) in which the input voltage is 1 V. For a general input voltage, Equation (11) provides a source level of the transducer.

(9) $$W=\frac{1}{2}{\left|U_{r}\right|}^{2}R_{r}$$

(10) $$D=\frac{{\left(k_{w}r_{h}\right)}^{2}}{1-J_{1}\left(2k_{w}r_{h}\right)/\left(k_{w}r_{h}\right)}$$

(11) TVR=10logW+10logD+170.8 (dB).

If *p* is the pressure at a far-field distance *r*_d_ from the sound source, the radiated acoustic power *П* transmitted by this source can be expressed as Equation (12) [29]. This acoustic power *П* can be equated to the acoustic power *W* evaluated from equivalent circuit analysis (ECA), as given in Equation (9), to obtain the radiated sound pressure *p*, as given in Equation (13). Then, Equation (13) can be used to analyze the sound pressure as a function of frequency while reflecting the effect of the structural parameters of the non-uniform drive section.

(12) $$\Pi =\frac{4\pi r_{d}^{2}P^{2}}{2\rho_{w}v_{w}}$$

(13) $$P=\sqrt{\frac{{\left|U_{r}\right|}^{2}R_{r}\rho_{w}v_{w}}{4\pi r_{d}^{2}}}.$$

## 3. Determination of the Circuit Parameters for the Multimode Vibration of the Head Mass

To complete the equivalent circuit for the multimode Tonpilz transducer, circuit parameters that represent the multimode vibration of the head mass (i.e., *M*_h1_, *M*_h2_, *M*_h3_, *C*_f_, and *R*_f_) should be determined. In this study, these equivalent circuit parameters were estimated with a fitness function, which was minimized through an unconstrained optimization process. The objective of the optimization was to minimize the fitness function by varying these unknown circuit parameters within a preset range. First, finite element analysis (FEA) was conducted for transient analysis of the simplified transducer in Figure 1 with the commercial software package PZFlex^®^. It is a two-dimensional axis-symmetric model. The finite elements were hexahedron-shaped with a size as small as λ/64 where λ is the wavelength at the highest frequency of analysis, which is 45 kHz. A water domain was constructed as the radiation medium having a sufficiently large size to provide the far-field distance. An absorbing boundary condition was applied all around the water domain to prevent any wave reflection at the outermost boundaries. The total number of elements in the model was about 44 thousand. The TVR spectrum was obtained through Fourier transform of the transient analysis result over the frequency range of 15–45 kHz at intervals of 100 Hz. The frequency range was selected to be sufficiently wide to include both the longitudinal and flexural mode resonance frequencies of the multimode Tonpilz transducer. The TVR levels at the flexural and longitudinal modes with their corresponding frequencies were referenced to define the fitness function. The fitness function (*DF*) was defined as the difference in TVR levels and their corresponding frequencies between the TVR spectra obtained from FEA and ECA:

(14) $$DF=\left|\frac{TVR_{L}-TVR_{L}^{\prime }}{TVR_{L}}\right|+\left|\frac{TVR_{F}-TVR_{F}^{\prime }}{TVR_{F}}\right|+\left|\frac{f_{L}-f_{L}^{\prime }}{f_{L}}\right|+\left|\frac{f_{F}-f_{F}^{\prime }}{f_{F}}\right|,$$

where TVR_L_ and TVR_F_ are the TVR levels from ECA in the longitudinal and flexural modes, respectively, while TVR′_L_ and TVR′_F_ are respectively those from FEA. The subscripts L and F indicate the longitudinal and flexural modes, respectively. The corresponding frequencies from ECA are *f*_L_ and *f*_F_, respectively, while those from FEA are f′_L_ and f′_F_, respectively. The fitness function *DF* was then minimized by varying the five circuit parameters. The circuit parameters with the best fit produced a TVR spectrum from ECA to match the spectrum from FEA most closely; these were identified as the correct circuit parameters to describe the multimode vibration of the head mass. Minimization was conducted with Opt-Quest Nonlinear Programming (OQNLP) algorithm, which is a multi-start heuristic algorithm that finds the global minimum of a constrained nonlinear problem [30].

**Figure 3:**
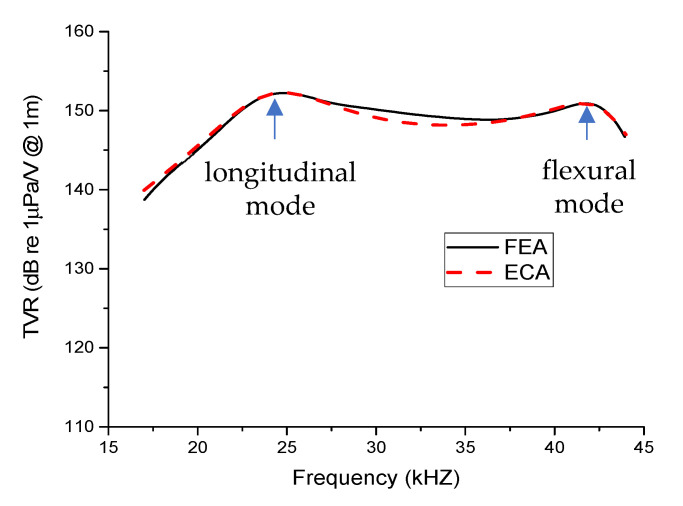
Comparison of the transmitting voltage response (TVR) spectra from finite element analysis (FEA) and equivalent circuit analysis (ECA) for the transducer with a uniform drive section.

The above circuit parameters were used to calculate the TVR spectrum of a multimode Tonpilz transducer. The results were compared with the FEA results. Table 2 lists the dimensions of the Tonpilz transducer model. Figure 3 shows the comparison for the transducer with a uniform drive section. The low-frequency peak corresponds to the longitudinal-mode resonance in which the whole transducer expands and contracts along the longitudinal direction whereas the high-frequency peak corresponds to the flexural-mode resonance which generates the bending of outer edges of the head mass [4,5,7].

**Figure 4:**
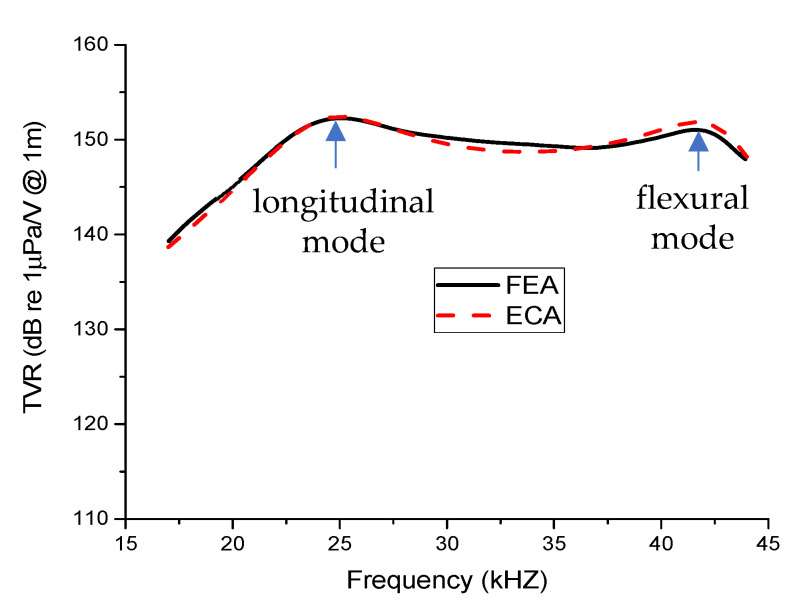
Comparison of the TVR spectra from FEA and ECA for the transducer with a non-uniform drive section.

The TVR spectrum for the multimode Tonpilz transducer with the uniform drive section from ECA using the circuits in Figure 2 showed good agreement with the FEA result. Figure 4 shows the same comparison for the transducer with a non-uniform drive section. The TVR spectra showed good agreement here as well. The estimated TVRs for longitudinal and flexural modes by the ECA were 152.4 dB and 151.8 dB, respectively, with a negligible difference of 0.1% and 0.5% from those by the FEA. Similarly, the difference in the resonance frequencies from the ECA and FEA was 0.3% for the longitudinal mode and merely 0.2% for the flexural mode. Thus, the estimated values for the circuit parameters *M*_h1_, *M*_h2_, *M*_h3_, *C*_f_, and *R*_f_ were confirmed to accurately predict the transmitting response of transducers with either a uniform or non-uniform drive section. These estimated values were utilized to investigate further the effects of varying the position and thickness of the lower, central, and upper stacks on the transmitting characteristics of the non-uniform Tonpilz transducer.

## 4. Analysis of the Effect of the PZT Stack Position in the Non-Uniform Drive Section

The advantage of the combined circuit in Figure 2 is that it allows the analysis of the effect of an individual stack on the performance of the transducer. In a Tonpilz transducer, the PZT stack is sandwiched between a lighter head mass and a heavier tail mass with a tail-to-head mass ratio of 2–4 [23]. The mass difference between the head and tail masses plays a critical role in a segmented PZT stack. The strain induced within the drive section varies with position; more strain occurs in the PZT stack near the tail mass [16]. In the present study, the effect of the stack position was investigated through an analysis of the sound pressure as different stacks in the drive section were excited. The analysis was conducted with the equivalent circuits derived in Section 3.

**Figure 5:**
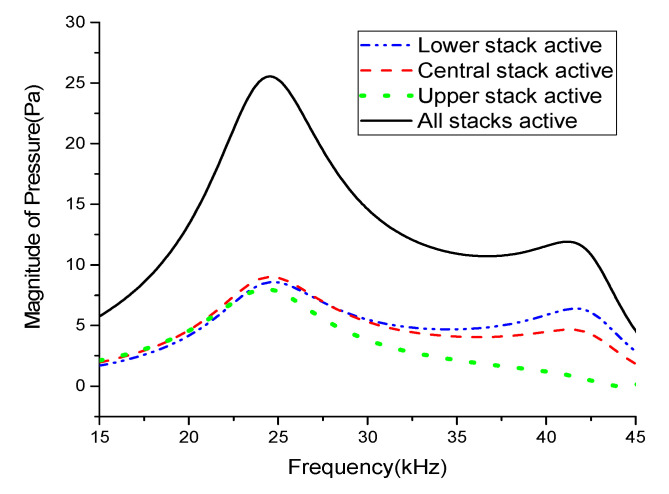
Comparison of the radiated sound pressures for individual and simultaneous excitation of the stacks in the uniform drive section.

First, ECA was performed for the multimode Tonpilz transducer with the uniform drive section to obtain the radiated sound pressures when the lower, central, and upper stacks were individually excited, as well as when the whole PZT stack was excited. Although there were no actual physical differences among the lower, central, and upper stacks for the uniform drive section, the drive section was divided into three stacks differing only in their relative position within the drive section, similar to the stacks in the non-uniform drive section. Figure 5 compares the radiated sound pressures for individual and simultaneous excitations of stacks in the uniform drive section. The quantitative sound pressures for the different excitation conditions are summarized in Table 3. The results indicate that the position of each stack had a significant effect. This effect increased for the flexural-mode frequency: the peak pressure reached its maximum with excitation of the lower stack and its minimum with excitation of the upper stack. When the lower stack was excited, the central and upper stacks acted as additional parts of the head mass. This heavier head mass increased the mechanical quality factor *Q*_m_ and resulted in a more prominent peak compared to the other two peaks as quantified in Table 3 [13,26]. In contrast, when the upper stack was excited, the lower and central stacks acted as additional parts of the tail mass. The heavier tail mass reduced *Q*_m_, which in turn resulted in an almost unnoticeable peak near the flexural-mode frequency [12,23]. In conclusion, the lower and middle stacks controlled the peak pressure magnitude near the flexural mode more effectively than the upper stack.

**Figure 6:**
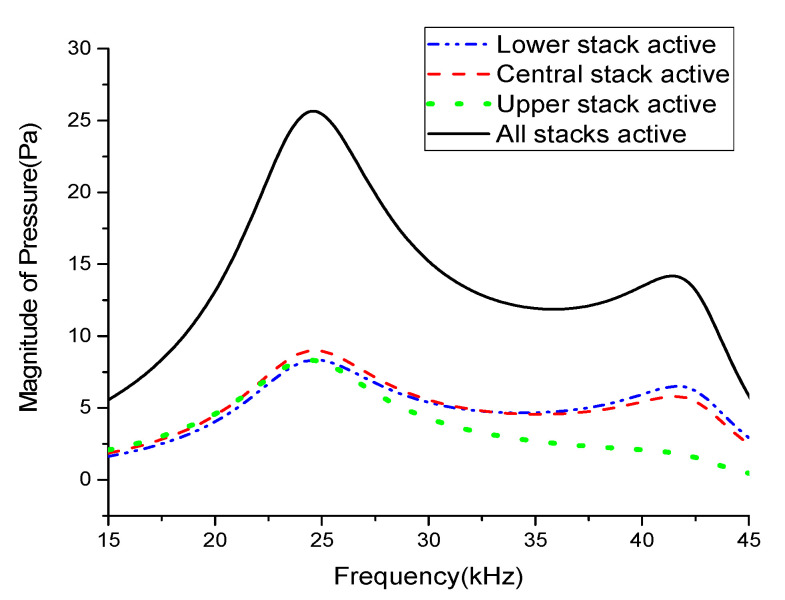
Comparison of the radiated sound pressures for individual and simultaneous excitation of the stacks in the non-uniform drive section.

In a similar way, the radiated sound pressures for individual and simultaneous excitations of the constituent stacks in the Tonpilz transducer with the non-uniform drive section were obtained through ECA and compared in Figure 6. The effect of the variations in the head and tail mass thicknesses caused by the relative position of the excited stack was also prominent. The peak pressure in the flexural mode reached its maximum when the lower stack was excited and its minimum when the upper stack was excited. However, the peak pressure magnitudes when the central and upper stacks were excited were higher with the non-uniform drive section than with the uniform drive section. This increase was attributed to the change in the relative position of the stacks caused by the non-uniform thicknesses. The central stack of the non-uniform drive section was approximately 2.66 mm lower than that of the uniform drive section, as given in Table 2. This change in position affected the distribution of the strain induced within each stack, which in turn increased the pressure magnitude, especially in the flexural mode [17].

**Figure 7:**
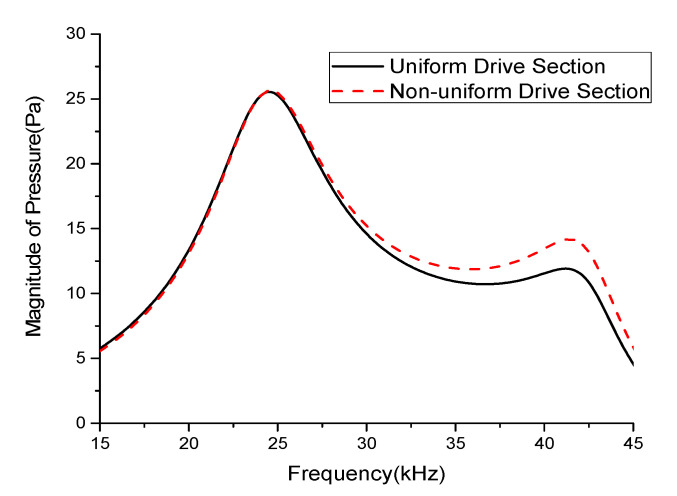
Comparison of the overall radiated sound pressures of Tonpilz transducers with uniform and non-uniform drive sections.

Table 3 summarizes the peak pressure magnitudes in the longitudinal and flexural modes for individual and simultaneous excitations of the stacks in the uniform and non-uniform drive sections. The relative position of the excited stack clearly had a significant effect on the radiated pressure magnitude, especially in the flexural mode. Figure 7 compares the total radiated sound pressures of the transducers with uniform and non-uniform drive sections. The radiated pressure for the multimode transducer with the non-uniform drive section was improved by 19.3% compared to that of the multimode Tonpilz transducer with a uniform drive section near the flexural mode resonant frequency. The longitudinal mode was affected by the whole length of the drive section, not by individual stacks, so the *TVR*_L_ remained almost constant regardless of the drive section structure. However, for the non-uniform drive section, the unequal thickness of stacks excited the higher frequency vibration more easily, which led to the higher *TVR*_F_ as observed in Figure 7. The improved radiation performance in the flexural mode can be utilized to increase the bandwidth of the multimode Tonpilz transducer.

## 5. Analysis of the Effect of the PZT Stack Thickness in the Non-Uniform Drive Section

In addition to the effect of position, the effect of the thickness of each PZT stack within the non-uniform drive section was investigated. The thicknesses of the lower, central, and upper PZT stacks were varied, while the overall thickness (*x*_0_) was kept constant at 20 mm.

**Figure 8:**
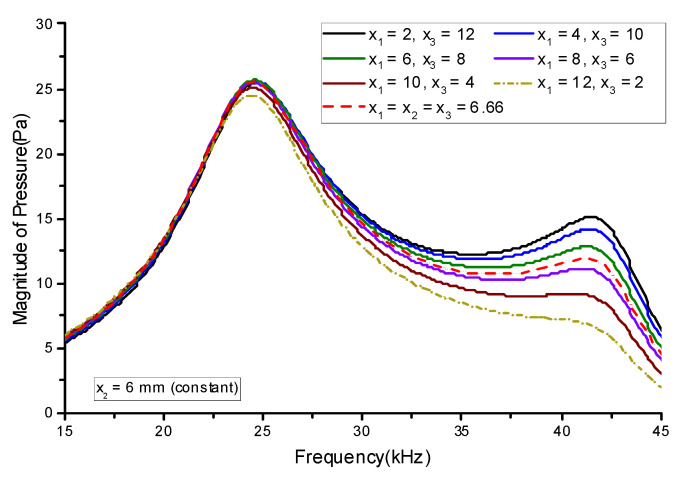
Effect of varying the lower and upper lead zirconate titanate (PZT) stack thicknesses on the sound pressure.

**Figure 9:**
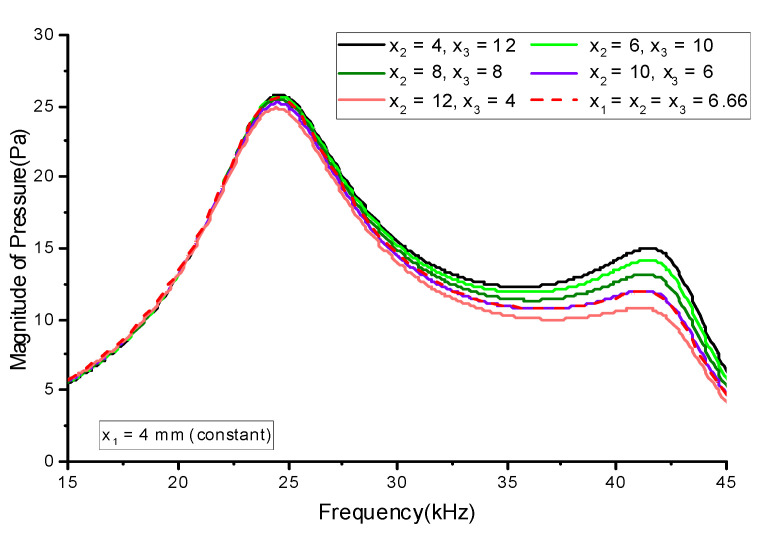
Effect of varying the central and upper PZT stack thicknesses on the sound pressure.

First, the thickness of the lower stack (*x*_1_) was varied from 2 to 12 mm in increments of 2 mm while the thickness of the central stack (*x*_2_) was kept constant at 6 mm. The thickness of the upper stack (*x*_3_) was varied in conjunction with *x*_1_ so that *x*_1_ + *x*_2_ + *x*_3_ = 20 mm. Thus, *x*_3_ was at its maximum when *x*_1_ was at its minimum and vice versa. The case when *x*_1_ was at its maximum was the same as if the initial lower stack and upper stack exchanged their positions. Figure 8 shows the effect of varying *x*_1_ in conjunction with *x*_3_ on the radiated sound pressure. Next, the effect of varying the central stack thickness *x*_2_ in conjunction with the upper stack thickness *x*_3_ was analyzed. *x*_2_ was varied from 4 to 12 mm in increments of 2 mm, and *x*_3_ was decreased to satisfy the condition *x*_1_ + *x*_2_ + *x*_3_ = 20 mm. Meanwhile, the lower stack thickness *x*_1_ was maintained at 4 mm. Figure 9 shows the effect of varying *x*_2_ in conjunction with *x*_3_ on the radiated sound pressure.

**Figure 10:**
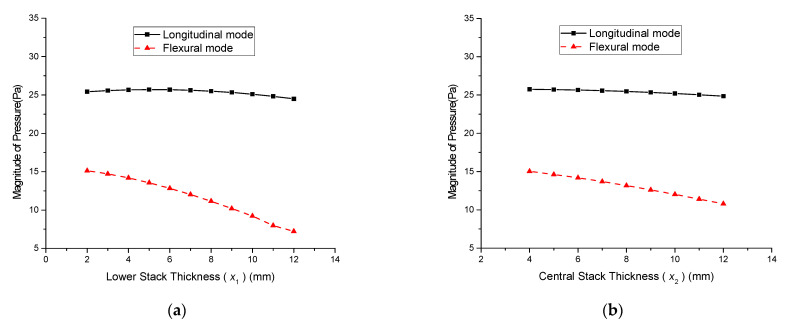
Variation in the sound pressure magnitude according to the thicknesses of the: (**a**) lower stack; (**b**) central stack.

Figure 8 shows that the magnitude of the sound pressure in both the longitudinal and flexural modes increased as *x*_1_ decreased. The increase in pressure was more prominent in the flexural mode. A similar trend for the sound pressure variation was observed when *x*_2_ was varied, as shown in Figure 9. Varying *x*_1_ had a more significant effect on the sound pressure than varying *x*_2_. Figure 10 summarizes the effect of varying the thickness on the sound pressure magnitude *p*. The pressure magnitude gradually decreased as the PZT stack thickness increased. Figure 10a indicates that the thinner stack should be at the bottom, like the initial geometry in Figure 1. For both cases in Figure 10, the pressure magnitude was reduced because of the proportional decrease in the electric field across the PZT stack (*E*_33_) as the stack thickness increased. Increasing the stack thickness reduced *E*_33_, which reduced the induced strain in the piezoceramic stacks and subsequently decreased the pressure magnitude. The decrease in the peak pressure magnitude was more prominent in the flexural mode. These results indicate that thinner lower and central stacks are more beneficial for enhancing flexural-mode vibrations. This can be utilized to increase the bandwidth of multimode Tonpilz transducers.

## 6. Experimental Validation of the Proposed Design Scheme

**Figure 11:**
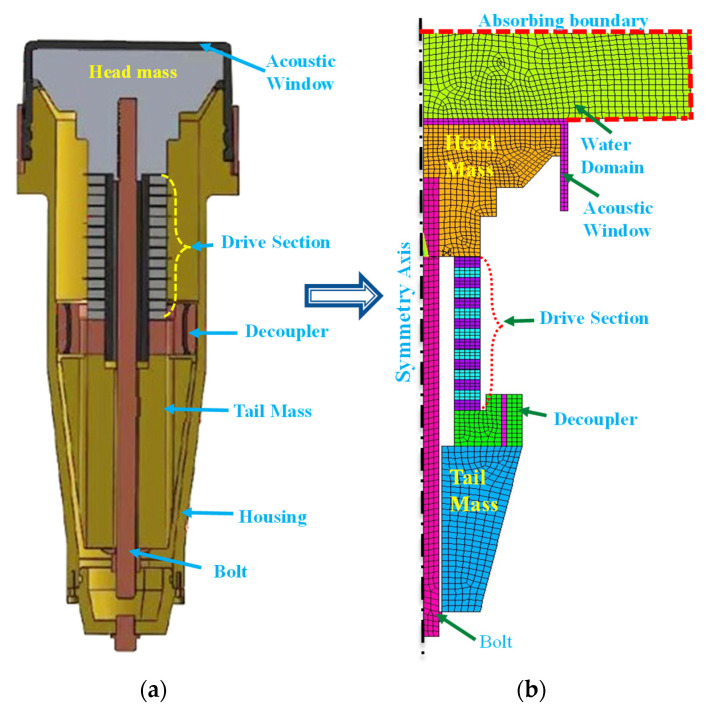
Structure of a practical multimode Tonpilz transducer: (**a**) cross-sectional view; (**b**) 2D axisymmetric model for FEA.

A prototype Tonpilz transducer was fabricated, and experiments were carried out to validate the proposed design scheme and confirm the effectiveness of the developed equivalent circuit. Figure 11a shows a schematic cross-sectional view of a practical Tonpilz transducer with a uniform drive section. In Figure 11a, the acoustic window provides impedance matching and structural sealing, and the decoupler isolates the transducer from the outer housing [4]. The main dimensions of the prototype transducer were as given in Table 4. The materials for most of the constituent components of the prototype transducer were the same as those presented in Table 1. The materials for the PZT disks, acoustic window, and decoupler were PZT-4, ethylene propylene diene monomer, and stainless steel, respectively. The material constants for these three components were taken from Afzal et al. [4] and Butler and Sherman [23].

To estimate the unknown circuit parameters corresponding to the flexural vibration mode, a simplified axisymmetric model of the prototype transducer was constructed as shown in Figure 11b. FEA was carried out to obtain the TVR spectrum in a similar manner as that described in Section 3. The equivalent circuit in Figure 2 was modified in accordance with the transducer structure in Figure 11b by the incorporation of an additional T-branch to include the effects of the acoustic window. Then, the equivalent circuit parameters were estimated, and ECA was conducted to obtain the TVR spectrum for the prototype transducer.

For the experimental validation, the individual components of the prototype transducer were fabricated according to the dimensions given in Table 4. These individual components were then assembled in a manner similar to that reported by Afzal et al. [4]. Measurements were carried out in a water tank with dimensions of 30 m × 15 m × 15 m. The prototype transducer was submerged to a depth of 6 m, and a hydrophone (Teledyne-RESON TC4032, Slangerup, Denmark) was placed at a far-field distance of 5.9 m from the prototype. The prototype transducer was excited with a power amplifier (Instruments Inc. L20, San Diego, CA, USA). The received hydrophone signal was amplified with a voltage amplifier (Teledyne-RESON EC6073, Slangerup, Denmark).

**Figure 12:**
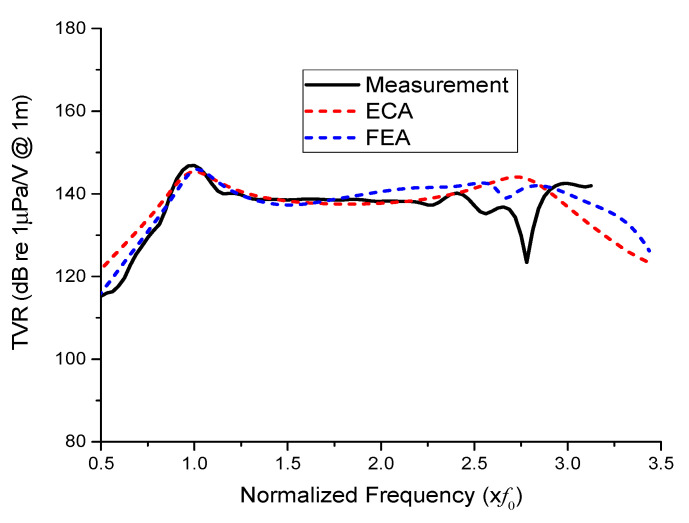
Comparison of the TVR spectra for the model with the uniform drive section obtained from FEA, ECA, and the experimental measurements.

Figure 12 compares the TVR results for the prototype transducer with a uniform drive section from the experimental measurement, ECA, and FEA. The TVR spectra were normalized with respect to the longitudinal resonant frequency (*f*_0_) of the transducer. In the longitudinal mode, the peak TVR levels and corresponding frequencies of the three TVR spectra showed very good agreement with each other. The measured peak TVR level was 146.8 dB; the difference from the ECA and FEA results was as negligible as 0.26% and 0.07%, respectively. The peak TVR frequencies were almost identical for the three cases. In the flexural mode, the resonant frequency and TVR level from the ECA were 2.71*f*_0_ and 144.0 dB, respectively. The flexural resonant frequency from the FEA was considered as the average of the two peaks before and after the slight dip in the TVR spectrum. The respective frequencies before and after the dip were 2.55*f*_0_ and 2.84*f*_0_ with corresponding TVR levels of 142.6 dB and 142.5 dB, respectively. Hence, the flexural mode resonant frequency and TVR level from the FEA were set to 2.70*f*_0_ and 142.6 dB, respectively. The difference between these resonant frequencies and TVR levels from the FEA and those from the ECA was considered to be mainly due to the simplification of the transducer structure in the ECA.

These TVR levels are slightly higher than that from the measurement, which was 140.2 dB. The peak TVR frequencies differed somewhat in the flexural mode. The difference was mainly attributed to the simplified structures in the analysis models and errors inherent to the experimental measurement. The effect of auxiliary components such as external housing and copper electrodes was not considered in the models for the sake of simplicity. The equivalent circuit also did not reflect the tapered geometry of the head and tail masses. Overall, however, the TVR spectra showed good agreement with each other.

**Figure 13:**
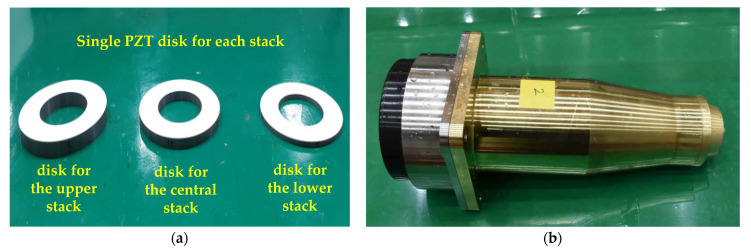
Photographs of the: (**a**) single PZT disks of different thicknesses for each stack, that is, upper, central, and lower stacks; (**b**) prototype transducer with the non-uniform drive section.

As the next step, a transducer structure was fabricated with a non-uniform drive section to verify that the non-uniform drive section improves the flexural mode vibration. The total length of the non-uniform drive section was 105 mm while the outer and inner radii of each PZT disk were 33 mm and 18 mm, respectively, which were the same as those of the uniform drive section. Each stack consisted of five disks. The thickness of each stack was set according to the trend analysis of thickness variation in Section 5. The final thicknesses of each stack were set to *x*_1_ = 12.6 mm, *x*_2_ = 24.4 mm, and *x*_3_ = 68.0 mm, which corresponded to the single disk thicknesses of each stack as *t*_c1_ = 2.5 mm, *t*_c2_ = 4.9 mm, and *t*_c3_ = 13.6 mm, respectively. The fabrication of a non-uniform drive section is difficult compared to that of the uniform drive section. Figure 13a shows the single PZT disks fabricated for the lower, central, and upper stacks, respectively. Because five of each single PZT disk constituted each stack, fifteen disks in total were assembled to form the non-uniform drive section by incorporating copper electrode layers between successive PZT disks in the configuration similar to that in Figure 1b. Figure 13b shows the final assembly of the prototype transducer with the non-uniform drive section and other structural components. The TVR spectrum of the multimode Tonpilz transducer with the non-uniform drive section was measured with the same equipment and electrical matching conditions as those for the transducer with the uniform drive section.

**Figure 14:**
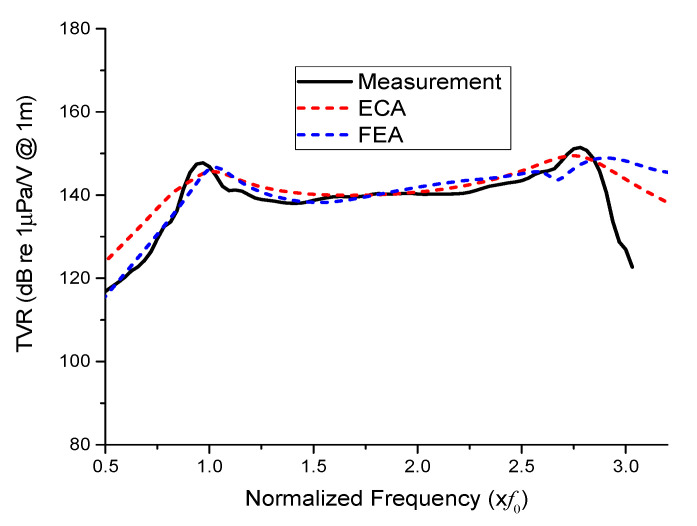
Comparison of the TVR spectra of the prototype Tonpilz transducer with the non-uniform drive section from ECA, FEA, and the experimental measurements.

Figure 14 compares the measured TVR spectrum of the prototype Tonpilz transducer with a non-uniform drive section against those estimated from ECA and FEA. Table 5 compares the acoustic performance. The peak TVR levels in the longitudinal and flexural modes and their corresponding frequencies showed good agreement between the measured and estimated values. The measured peak TVR level was 147.8 dB in the longitudinal mode; this was higher than the FEA and ECA results by 1.1 dB and 2.1 dB, respectively. Similarly, the measured peak TVR level of the flexural mode was 151.4 dB, which was higher than the FEA and ECA results by 2.5 dB and 1.9 dB, respectively. The measured frequency was 1.1% higher than the ECA result in the flexural mode but 4.9% lower in the longitudinal mode.

**Figure 15:**
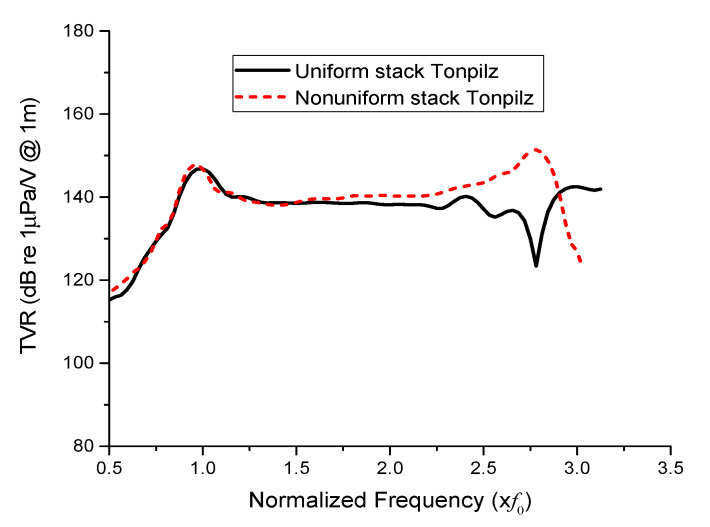
Comparison of the TVR spectra of the prototype Tonpilz transducer with uniform and non-uniform drive sections.

The discrepancies in the longitudinal- and flexural-mode TVR levels and their corresponding frequencies between the three evaluation methods were attributed to the simplified structures for ECA and FEA as well as the fabrication tolerances for the prototype transducer. However, the objective of the experiment was to verify the efficacy of the non-uniform drive section in improving the flexural mode vibration of the transducer. In the flexural vibrational mode, the non-uniform drive section resulted in a TVR level of 151.4 dB, while the uniform drive section resulted in a TVR level of 140.2 dB. Thus, the non-uniform drive section clearly improved the acoustic performance in the flexural mode, which can improve the wideband characteristics of the Tonpilz transducer as shown in Figure 15. In comparison with the authors’ previous work on multimode Tonpilz transducers [4], the bandwidth between the longitudinal and flexural resonance frequencies increased from 1.45*f*_0_ to 1.81*f*_0_, which is 24.8% increase. This demonstrates the efficacy of the proposed design scheme. The proposed equivalent circuit can be conveniently extended to Tonpilz transducers with non-uniform drive sections having more stacks and an arbitrary number of PZT disks in each stack.

## 7. Conclusions

In this study, a simple structural method was proposed to improve the transmitting characteristics of a multimode Tonpilz transducer. Compared with other existing methods that require a complex geometric shape or additional components, the proposed method simply incorporates a non-uniform drive section comprising PZT stacks of different thicknesses without modifying any other components of the transducer. The non-uniform drive section enhanced the flexural vibration mode of the transducer, which could eventually widen the bandwidth of the transducer. The design of the non-uniform drive section is a little more complicated than that for the uniform drive section, but the design is much simpler than designing a whole new structure of a wideband multimode Tonpilz transducer. A new equivalent circuit was developed to represent the non-uniform structure of the drive section, where the response of the whole drive section could be determined by superposing the responses of constituent circuits. Each constituent circuit corresponded to a stack of different thicknesses in the non-uniform drive section. The validity and efficacy of the design scheme was verified through the fabrication and characterization of a prototype multimode Tonpilz transducer.

This work can be extended to structural optimization of the non-uniform drive section. The proposed equivalent circuit can be used to obtain the wideband characteristics of multimode Tonpilz transducers in a much more efficient manner than by conventional methods such as the FEM. The developed structure can be readily extended to an arbitrary number of stacks in the Tonpilz transducer with any number of PZT disks in each stack.

## Figures and Tables

**Table 1:** Material properties of the components of the Tonpilz transducer.

Model Section	Material	Poisson’s Ratio	Density (kg/m^3^)	Young’s Modulus (GPa)	Sound Velocity (m/s)
Head mass	Aluminum	0.34	2700	71	6148
Tail mass	Brass	0.35	8800	120	4184
Bolt	Alloy steel	0.28	8250	210	4570
Acoustic window	Urethane	0.43	1065	0.97	1284
Radiation medium	Water	-	1000	-	1500

**Table 2:** Dimensions of the simplified multimode transducer model.

Structural Parameter	Dimension (mm)	Structural Parameter		Dimension (mm)
Head mass radius (*r*_h_)	21.0	Inner radius (*r*_i_)		2.8
Head mass thickness (*h*_h_)	11.0	Single disk/stack thickness for the uniform drive section	*t*_c0_/*x*_0_	3.3/6.7
Tail mass radius (*r*_t_)	15.0			
Tail mass thickness (*h*_t_)	14.0	Single disk/stack thickness for the non-uniform drive section	*t*_c1_/*x*_1_	2.0/4.0
Cap thickness (*h*_hc_)	4.0		*t*_c2_/*x*_2_	3.0/6.0
PZT stack radius (*r*_c_)	12.0		*t*_c3_/*x*_3_	5.0/10.0

**Table 3:** Comparison of the peak pressure magnitudes (unit: Pa).

Drive Section Structure	Excited Stack	Longitudinal Mode	Flexural Mode
Uniform	Lower stack	8.6	6.4
	Central stack	9	4.7
	Upper stack	8	1
	All stacks	25.5	11.9
Non-uniform	Lower stack	8.4	6.5
	Central stack	9	5.8
	Upper stack	8.3	1.9
	All stacks	25.7	14.2

**Table 4:** Main dimensions of the prototype multimode Tonpilz transducer.

Structural Parameter	Dimension (mm)	Structural Parameter	Dimension (mm)
Head mass radius (*r*_h_)	80	Acoustic window thickness	3
Head mass thickness (*h*_h_)	90	PZT stack outer radius (*r*_c_)	33
Tail mass outer radius (*r*_t_)	55.5	PZT stack inner radius	18
Tail mass thickness (*h*_t_)	140	Single PZT disk thickness	7
Inner radius of head and tail masses	10	Total number of PZT disks	15

**Table 5:** Comparison of the longitudinal- and flexural-mode performances according to ECA, FEA, and the measurement.

Evaluation Method	Longitudinal- Mode TVR (dB)	Flexural-Mode TVR (dB)	Longitudinal Resonance Frequency	Flexural Resonance Frequency
ECA	145.7	149.5	1.02*f*_0_	2.75*f*_0_
FEA	146.7	148.9	1.02*f*_0_	2.89*f*_0_
Measurement	147.8	151.4	0.97*f*_0_	2.78*f*_0_

## Data Availability

Not applicable.
